# Accelerating the spread of laboratory quality improvement efforts in Botswana

**DOI:** 10.4102/ajlm.v3i2.207

**Published:** 2014-11-03

**Authors:** Kelebeletse O. Mokobela, Mpho T. Moatshe, Mosetsanagape Modukanele

**Affiliations:** 1 Ministry of Health, Botswana; 2 US Centers for Disease Control and Prevention, Botswana

## Abstract

**Background:**

In 2002, the Ministry of Health (MoH) of Botswana began its journey toward laboratory accreditation in an effort to enhance the quality of laboratory services. After a difficult start, the MoH recognised the need for a more practical and sustainable method for change that could be implemented nationally; they therefore adopted the Strengthening Laboratory Management Toward Accreditation (SLMTA) programme.

**Objective:**

This study describes the process and lessons learned in implementing SLMTA and the role of supplemental training and mentoring so as to achieve Botswana’s national laboratory quality improvement goal.

**Methods:**

Eight laboratories were enrolled into the SLMTA programme in 2010, which included a series of workshops and improvement projects conducted over nine months. Four of these laboratories received supplementary training and focused mentorship from the Botswana Bureau of Standards (BOBS). Laboratory performance was measured at baseline and exit using the World Health Organization Regional Office for Africa’s Stepwise Laboratory Quality Improvement Process Towards Accreditation (SLIPTA) checklist. One laboratory did not receive an exit audit and was thus excluded from the analysis.

**Results:**

An 18 percentage-point improvement was observed when comparing the median baseline score (53%) to the median exit score (71%) for the seven laboratories. Laboratories that received additional training and mentorship from BOBS improved 21 percentage points, whilst non-BOBS-mentored laboratories improved eight percentage points. Hospital management buy-in and strong laboratory staff camaraderie were found to be essential for the positive changes observed.

**Conclusion:**

SLMTA facilitated improvements in laboratory quality management systems, yielding immediate and measurable results. This study suggests that pairing the SLMTA programme with additional training and mentorship activities may lead to further increases in laboratory performance; and that SLMTA is a practical approach to extending quality improvement to MOH laboratories.

## Introduction

Laboratory quality management systems (QMS) provide a strong foundation for promoting excellence in laboratory services that support fundamental components of effective healthcare systems.^1^ In many resource-limited countries, laboratories lack robust quality systems, as they have historically been afforded low priority and few resources. This situation has led to poor-quality patient care and health outcomes, as well as loss of revenue resulting from inefficient and redundant processes. However, in recent years, an increased focus has been placed on the delivery of quality services as governments have moved toward initiating improvements in laboratory services.^1,2^

In 2002, the Botswana Ministry of Health’s (MoH’s) Laboratory Services developed a five-year work plan with the goal of accrediting 16 laboratories, which resulted in the initial introduction of QMS in select laboratories. Six years later, limited improvements in the quality of laboratory management and service were noted because of high workload, inadequate staffing and poor infrastructure, amongst other factors. By 2011, after nine years of implementation and extensive partner and consultant support, four laboratories had attained international accreditation. Whilst this accomplishment was commendable, it had been clear for some time that this approach was too costly (as consultants from outside Botswana were employed) and too slow to be a sustainable option for long-term quality improvement on a national level; Botswana needed a more viable strategy.

In 2008, the MoH developed a five-year (2009–2014) National Laboratory Strategic Plan,^3^ which called for implementation of QMS in all laboratories by 2014 and accreditation of four district-level and two national-level laboratories by 2013 and 2014, respectively. The strategic plan directed country laboratory QMS activities by incorporating a mentoring approach for laboratory accreditation. Shortly after initiating this plan, the MoH adopted the Strengthening Laboratory Management Toward Accreditation (SLMTA) programme so as to catalyse the operation of the strategic plan and provide a platform to promote quality management of laboratories. In accordance with the strategic plan, key laboratories throughout the country were identified to participate in the SLMTA programme, which consists of a comprehensive management framework, training and mentoring toolkit, and a multi-workshop implementation model.^4^

Whilst the SLMTA programme formed the cornerstone of Botswana's laboratory improvement strategy, the MoH theorised that combining SLMTA with additional QMS training and targeted mentoring might achieve superior results.^5^ Therefore, additional training and mentorship were offered to four top-priority laboratories designated as future Centres of Excellence. This article describes the process and lessons learned in implementing SLMTA and the role of supplemental training and mentoring so as to achieve Botswana's national QMS and accreditation goals.

## Research methods and design

### The SLMTA programme

The MoH enrolled eight national, regional, district and primary level laboratories throughout Botswana in the SLMTA programme, beginning in August 2010. Profiles of each laboratory (A to H) are listed in Table 1. Implementation followed the standard SLMTA process with a series of three workshops delivered over a period of nine months.^5^ A total of 24 laboratory staff, including laboratory managers, quality officers and section heads, participated in the training.

**TABLE 1:** Profiles of laboratories enrolled in the Botswana SLMTA programme, 2010.

Code	Laboratory	Level	Tests provided	Number of staff enrolled in SLMTA/total number of staff
A	National Health Laboratory	National	Special Chemistry, Histopathology, Cytopathology, Public Health Microbiology	5/26
B	Princess Marina Hospital Laboratory	Regional	Chemistry, Haematology, Microbiology, Blood Banking	3/40
C	Thamaga Primary Hospital Laboratory	Primary	Chemistry, Haematology, Microbiology, Blood Banking and CD4	2/5
D	Selebi-Phikwe Hospital Laboratory	District	Chemistry, Haematology, Microbiology, Blood Banking, Viral Load and CD4	2/13
E*	Sekgoma Memorial Hospital Laboratory	District	Chemistry, Haematology, Microbiology, Blood Banking, Viral Load and CD4	3/20
F*	Mahalapye Hospital Laboratory	District	Chemistry, Haematology, Microbiology, Blood Banking, Viral Load and CD4	3/14
G*	Letsholathebe II Memorial Hospital Laboratory	District	Chemistry, Haematology, Microbiology, Blood Banking, Viral Load and CD4	3/19
H*†	Scottish Livingstone Hospital Laboratory	District	Chemistry, Haematology, Microbiology, Blood Banking, Viral Load and CD4	3/24

* Mentored by the Botswana Bureau of Standards (BOBS).

† Excluded from analysis as a result of missing exit audit data.

SLMTA, Strengthening Laboratory Management Toward Accreditation.

Each workshop was followed by a period of three months to allow participants to implement improvement projects. Laboratory staff members were allowed to choose improvement projects that were relevant to their local environment and priorities. Each laboratory was encouraged to involve all laboratory staff members in improvement project implementation.

After each workshop, two follow-up visits were conducted by MoH/SLMTA trainers in order to provide further training and coaching on improvement projects. The trainers spent one day in each laboratory. The visits included meetings with hospital management so as to create awareness and solicit support for the laboratory improvement process.

### Additional training and mentorship in selected laboratories

Four of the eight laboratories (E, F, G and H) had been recently relocated to new facilities designated as Centres of Excellence in medical specialties. These facilities received additional training by the Botswana Bureau of Standards (BOBS), a certified International Organization for Standardization (ISO) training organisation. Training focused on understanding the auditing and documentation requirements for ISO 15189. BOBS also provided extra mentoring to these four laboratories from April to June 2011; monthly visits lasted one week in each laboratory. BOBS provided mentorship on system documentation, covering the development of quality manuals, standard operating procedures (SOPs) and other quality documents as required by the ISO standard. BOBS mentors, together with the laboratory staff, conducted a gap analysis and developed a work plan with deliverables for the mentee laboratories. Once a task from the work plan was completed (e.g., writing a quality manual), the laboratory would share it with the mentor who would then make corrections. The mentor provided guidance on the document’s layout, as well as on interpretation of different clauses of the ISO standard. After finalising the documents, the mentor assisted with implementation. The laboratory and the mentor established a relationship that allowed both parties to communicate via email or telephone, as needed, between visits.

### Evaluation

SLMTA in-country trainers conducted audits in order to measure laboratory improvements using the World Health Organization Regional Office for Africa’s Stepwise Laboratory Quality Improvement Process Towards Accreditation (SLIPTA) checklist.^6^ The SLIPTA checklist is organised around the 12 Quality System Essentials (QSEs).^7^ For each QSE, a score is obtained by calculating the points that a laboratory has received from each item on the checklist. An overall score is used to rate laboratories on a zero- to five-star rating scale, with a score of < 55% as zero stars, 55% – 64% as one star, 65% – 74% as two stars, 75% – 84% as three stars, 85% – 94% as four stars and ≥ 95% as five stars. SLIPTA was used for both the baseline (July 2010) and exit (November 2011) audits.

From July 2012 to February 2013, BOBS mentors conducted trial assessments in their laboratories using the South African National Accreditation System’s (SANAS) checklist in order to determine the laboratories’ readiness for accreditation by SANAS, a key regional accreditation body for southern Africa.^9^ This checklist yields qualitative results rather than a numerical score and highlights areas of focus for accreditation preparation.

One of the eight laboratories (Laboratory H) did not receive an exit audit as a result of a schedule conflict and therefore is not included in the SLIPTA data analysis in this article; it did, however, participate in the trial assessment using the SANAS checklist.

### Results

**FIGURE 1:**
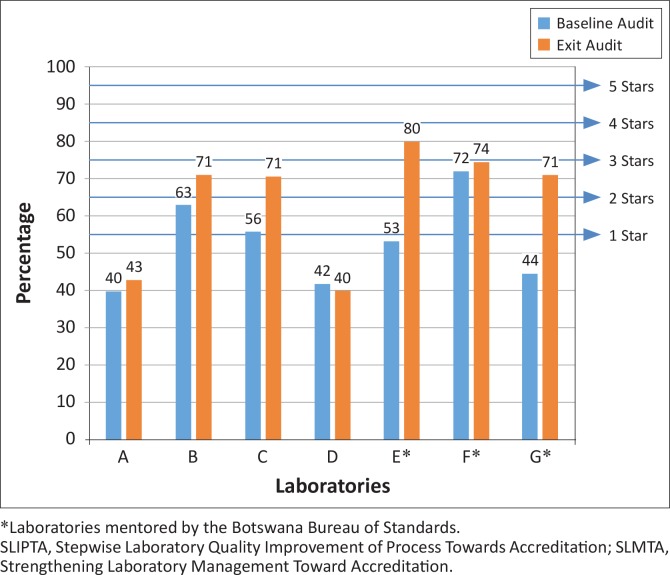
SLIPTA scores and star levels at the baseline and exit audits, Botswana SLMTA programme 2010–2011.

At baseline, four of the seven laboratories had a zero-star rating, two had one star and one had two stars. At exit, two laboratories remained at zero stars, four laboratories were rated at two stars and one laboratory was rated at three stars. The median SLIPTA audit scores were 53% at baseline and 71% at exit, a median increase of 18 percentage points. The three BOBS-mentored laboratories improved by 21 percentage points from a median score of 53% at baseline to 74% at exit, whilst the non-BOBS-mentored laboratories increased eight percentage points from a median score of 49% at baseline to 57% at exit. The greatest improvements were in two of the BOBS-mentored laboratories (E and G), which gained 27 percentage points each. Among non-BOBS-mentored laboratories, one (D) had a slight decrease in score, whilst the others each increased by three to 15 percentage points (Figure 1).

**FIGURE 2:**
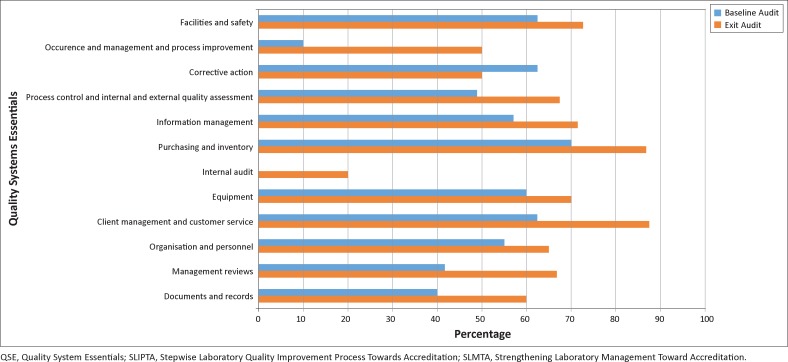
Mean QSE scores at the baseline and exit audits, Botswana SLMTA programme 2010-2011 (*n* = 7).

Comparing the median performance scores at baseline with those at exit for each QSE across the seven laboratories, the greatest improvement was in occurrence management and process improvement (40 percentage points), management reviews (25 percentage points), client management and customer service (25 percentage points), documents and records (20 percentage points) and internal audit (20 percentage points). Corrective action, however, had a 13 percentage-point drop from baseline to exit (Figure 2).

**FIGURE 3:**
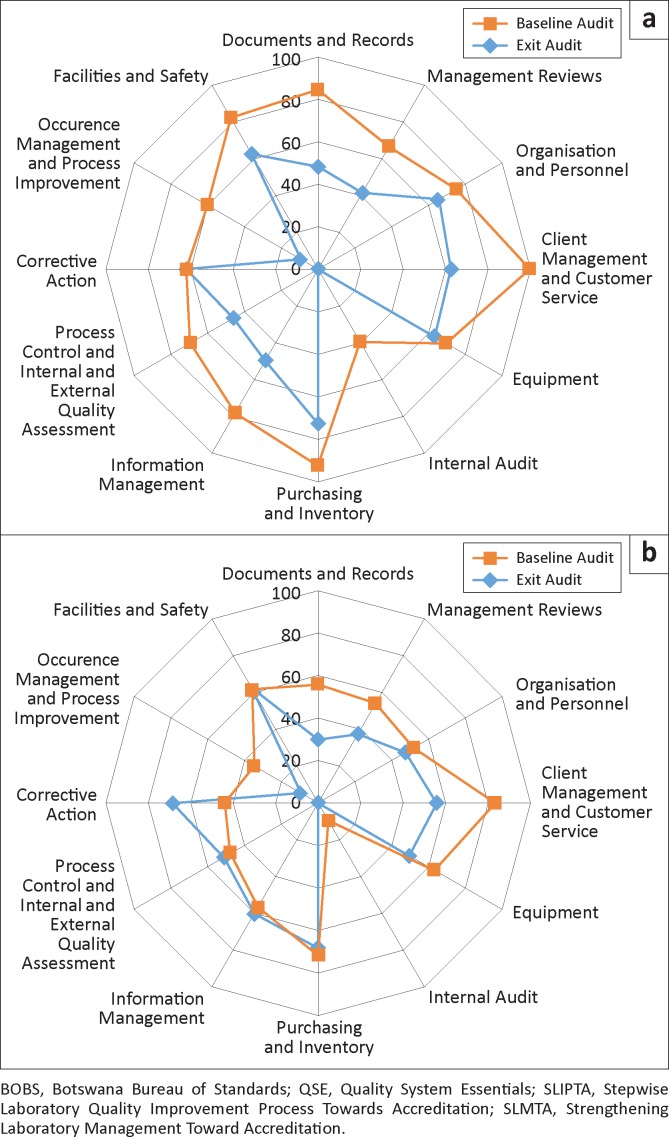
Mean QSE scores of (a) BOBS-mentored laboratories (*n* = 3) and (b) non-BOBS-mentored laboratories (*n* = 4) at the baseline and exit audits, Botswana SLMTA programme 2010−2011.

Figure 3a shows the performance of the three BOBS-mentored laboratories (E, F and G) in the 12 QSE sections of the SLITPA checklist. For these laboratories, the greatest gains were in the areas of occurrence management and process improvement (50 percentage points), internal audit (40 percentage points), client management and customer service (38 percentage points) and documents and records (36 percentage points). For the non-BOBS-mentored laboratories (A, B, C and D), documents and records (26 percentage points) and client management and customer service (25 percentage points) had the greatest improvement (Figure 3b). None of the QSE scores dropped for BOBS-mentored laboratories; for non-BOBS laboratories, however, scores dropped in three areas: corrective action (25 percentage points); information management (4 percentage points); and process control and internal and external quality assessment (3 percentage points).

In the trial assessment using the SANAS checklist after the exit audit in the BOBS-mentored laboratories (E, F and G), the laboratories had made additional improvements in many areas (Table 2). For example, quality manuals were now complete, internal audits were executed and safety procedures were in place in most of the audited laboratories. Some notable deviations included: incomplete finalisation of critical documents; out-of-date and incomplete equipment services and calibrations; and incomplete external quality assessment (EQA) programmes. Following this audit, Laboratory (E) and Laboratory (H) were encouraged to apply for international accreditation.

**TABLE 2:** Qualitative results from trial assessment using the SANAS checklist in BOBS-mentored laboratories enrolled in the Botswana SLMTA programme 2010−2011.

Area of observation	Laboratory E	Laboratory F	Laboratory G	Laboratory H
Quality manual	Available and authorised.	Available and authorised, however lacking in some essential elements.	Available and authorised.	Available, authorised and distributed.
Technical procedures	Developed and distributed to all sections.	Developed and distributed to all sections. Some SOPs were not controlled. Some procedures need finalisation. Method validation performed for all tests except viral load.	Completed for some of the testing areas.	Developed and distributed to all sections. Challenges in method validation.
EQA	Performed on all tests except for malaria, creatinine and cholesterol. Performance of EQA acceptable.	Performed on all tests except haematology. Performance of EQA acceptable.	Performed on all tests; evaluation of results commenced, but with delays.	Performed on all tests with satisfactory results.
Staff competence	Staff competency records available.	No records available.	Procedure available but not implemented.	Staff competency records available.
Internal audits	Procedures and policies available. Audits conducted by trained personnel; however no clear timelines identified on closure of nonconformities.	Procedure and policy available. Audits conducted by trained personnel.	Conducted only in the Chemistry section.	Two internal audits performed per year by trained auditors. Nonconformities closed at the time of audit.
Management review	Scheduled once a year but were not conducted at the time of audit.	Scheduled for once a year and are being conducted.	Procedure available but review was not conducted.	Four meetings planned annually and one was conducted.
Equipment maintenance and calibration	Maintenance and calibration in place, though not completed for all equipment.	Maintenance and calibration plans in place.	Equipment calibration in place but not fully implemented. Internal checks done and records maintained.	Equipment maintenance programmes in place but not fully implemented. Internal checks and records in place.
Safety	Safety manual available.	Safety manual in place and distributed.	Safety manual available. Training needed for safety officers. Hepatitis B vaccination programme in place; however, cleaners not vaccinated.	Safety manual in place and distributed.
General observations	QMS developed and implemented accordingly. Laboratory ready to apply for accreditation.	QMS developed and implemented accordingly; however, major gaps were identified. Laboratory to close identified gaps before applying for accreditation.	Laboratory will address identified nonconformities in view to consideration for applying for accreditation. Implementation of the QMS to be verified.	QMS developed and implemented accordingly. Laboratory ready to apply for accreditation.

SANAS, South African National Accreditation System; BOBS, Botswana Bureau of Standards; SLMTA, Strengthening Laboratory Management Toward Accreditation; EQA, External Quality Assessment; SOP, Standard Operating Procedure; QMS, Quality Management System.

## Discussion

The introduction of the SLMTA programme in hospitals in Botswana was found to be a practical option that yielded positive results for strengthening laboratories. All but one laboratory demonstrated improvements and, as a whole, laboratories improved in all QSEs except corrective action.

Supplemental mentorship and training may have contributed to the success amongst BOBS-mentored laboratories, which showed greater median improvements in the SLIPTA audit results. However, it is important to note that the small number of laboratories and lack of random assignment to BOBS mentorship limits the ability to draw definitive conclusions regarding this comparison. Other studies have also noted that supplementing SLMTA with additional training and mentoring may be beneficial.^9,10,11^ Our findings, when joined with these others, suggest that combining SLMTA with other strategies in a QMS programme may lead to synergistic improvement.

One of the keys to success of the roll-out of SLMTA in the laboratories was strong staff commitment and involvement. During the training sessions, staff involvement was cultivated by the formation of teams that brainstormed improvement projects and outlined specific implementation tasks. This practice fostered a culture of problem solving and boosted confidence amongst laboratory staff, who felt empowered to implement improvement projects previously considered beyond their capability. These projects were developed by the trainees and responsibility was shared across the laboratory team. Laboratories showed improvements in areas in which they had previously struggled, such as internal audit and management reviews. Some laboratories also improved their inventory control systems and anecdotally reported decreased turnaround times.

Support and buy-in from hospital management for SLMTA was another critical component of the quality improvement process. As understanding and ownership of the process increased amongst hospital management, managers became more willing to assist laboratories in improvement projects that required additional funds or involvement from multiple hospital departments. For example, some projects required infrastructure modifications; hospital management provided resources for sink relocations, trunks to hold electrical cords and facility painting. One hospital hired extra temporary staff to assist with phlebotomy duties whilst laboratory staff concentrated on quality improvement activities. As a result, laboratory staff morale and commitment improved.

Three laboratories had minimal improvements or decreased scores. Laboratory A, the National Health Laboratory, serves multiple functions: as a national reference laboratory, a procurement agency and a training agency. Balancing the requirements of these diverse functions was a unique challenge that will require resolution before tangible results can be achieved. Laboratory F had initially shown improvements, but had difficulties in getting documents reviewed and authorised by laboratory management prior to the exit audit. In Laboratory D, both of the SLMTA-trained staff members transferred to other facilities during the implementation of the programme, which impacted negatively on performance.

Limited progress was observed across all the laboratories in the areas of equipment; facilities and safety; and organisation and personnel, indicating that further work is needed. These areas may require greater funding and management involvement at higher levels, which the MoH is attempting to address. For example, throughout the country medical equipment maintenance and calibration programmes are weak and national EQA programmes and laboratory information systems do not meet the needs of laboratories.

As the five-year National Laboratory Strategic Plan comes to an end, the MoH is in the process of launching a follow-up plan for 2015 to 2019. This plan seeks to address remaining challenges and to identify ways of maintaining and increasing the improvement that was achieved in all participating laboratories. One new approach will be to use the many documents (e.g., SOPs and safety manuals) developed during the SLMTA programme as the standard national documents for distribution to all MoH laboratories. In addition, to ensure sustainability and continuous quality improvement amidst reduced donor funding, an additional 18 local mentors have been trained to help accelerate the spread of laboratory quality improvement efforts.

### Conclusion

The effort for widespread improvement of laboratory services in Botswana has gained momentum in the past few years following the introduction of the SLMTA programme. The programme was well received by staff for its practicality and measurable impact, as improvement was demonstrated in most of the enrolled laboratories. Whilst positive gains have been achieved, progress still needs to be made in struggling areas to minimise system disruptions in the laboratory and improve the national service programmes that support them. This study adds to the growing body of evidence that a combined strategy of SLMTA plus targeted training and mentorship can lead to further effectiveness of a QMS programme and higher-quality laboratory service delivery.
